# Molecular Insight into Affinities of Gallated and Nongallated Proanthocyanidins Dimers to Lipid Bilayers

**DOI:** 10.1038/srep37680

**Published:** 2016-11-22

**Authors:** Wei Zhu, Le Xiong, Jinming Peng, Xiangyi Deng, Jun Gao, Chun-mei Li

**Affiliations:** 1 College of Food Science and Technology, Huazhong Agricultural University, Wuhan, 430070, China; 2 Hubei Key Laboratory of Agricultural Bioinformatics, College of Informatics, Huazhong Agricultural University, Wuhan, 430070, China; 3 Key Laboratory of Environment Correlative Food Science, Ministry of Education, Huazhong Agricultural University, Wuhan, 430070, China.

## Abstract

Experimental studies have proved the beneficial effects of proanthocyanidins (Pas) relating to interaction with the cell membrane. But the detailed mechanisms and structure-function relationship was unclear. In present study, molecular dynamics (MD) simulations were used to study the interactions of four PA dimers with a lipid bilayer composed of 1:1 mixed 1-palmitoyl-2-oleoyl phosphatidylcholine (POPC) and 1-palmitoyl-2-oleoyl phosphatidylethanolamine (POPE). The results showed that the gallated PA dimers had much higher affinities to the bilayer with lower binding free energies compared with nongallated PA dimers. The gallated PA dimers penetrated deeper into the bilayer and formed more hydrogen bonds (H-bonds) with bilayer oxygen atoms, especially the deeper oxygen atoms of the lipids simultaneously, thus inducing stronger lateral expansion of the membrane and lipid tails disorder. The present results provided molecular insights into the interactions between PA dimers and bio-membranes and agreed with our experimental results well. These molecular interactions helped to elucidate the structure-function relationship of the PA dimers and provided a foundation for a better understanding of the underlying mechanisms of the bioactivities of PA oligomers.

Proanthocyanidins (PAs) are a group of polyphenolic compounds with a broad range of health-promoting effects, such as anticarcinogenic, antimicrobial, antioxidant, anti-hyperlipidemia and anti-inflammatory activity^1^,^2^,^3^,^4^. However, the biological activity of PAs differs greatly due to the difference in structure. For instance, catechins with a pyrogallol-type structure in the B-ring showed significant apoptosis inducing effect on human histiocytic lymphoma U937 cells, and an additional 3-O-gallate group in the B ring enhanced the activity, while catechins without a pyrogallol-type structure showed no apoptosis inducing activity^5^,^6^,^7^. Similarly, *cis*-type epigallocatechin-3-gallate (EGCG) was reported to have significantly higher apoptosis-inducing activity than its corresponding *trans*-type gallocatechin-3-gallate (GCG) in the same cultured cells^8^. These results indicated that small differences in the structure of PAs will result in significantly different bioactivities.

PAs can be classified into monomers (DP = 1), oligomers (DP = 2–10) and polymers (DP > 10) based on the degree of polymerization (DP)^9^. Among which, PA monomers and oligomers attracted more attention due to the higher concentration in food and more potent bioactivity than polymers *in vivo*^10^. As a typical representative of PA monomer, catechins attracted the greatest interest world-wide. Over the last decades, studies on the biological potentials, as well as the structure-activity relationship of catechins, are highlighted. For example, epicatechin-3-gallate (ECG) and EGCG were reported to exert more pronounced antioxidant and antibacterial effects than catechin (C) and epicatechin (EC) in a liposome system *in vitro*^11^. Additionally, ECG, EGCG and theaflavin-3-gallate were reported to display stronger anti-cancer effects in HT29 colon cancer cells than EC^12^. Manabu *et al*. also reported the catechin-3-gallate (CG), EGCG, but not catechins EC and EGC, inhibit insulin-induced translocation of glucose transporter (GLUT4) by the insulin signaling pathway in 3T3-L1 cells^13^. Although some authentic conclusions including that gallated catechins always showed higher activities than non-gallated catechins, and *cis*-type catechins were more potent than the corresponding *trans*-type ones can be obtained from previous studies^13^,^14^, the detailed mechanisms are poorly elucidated. Compared with PA monomers, the structures of PA oligomers are more complex. It is known that the bioactivity of PA oligomers is highly structure-dependent and the chemical structures, such as monomer compositions, the linkage type of interflavan bonds and the DP, largely affect the bioactivities of PAs^15^,^16^. However, limited studies on the structure-activity relationship of PA oligomers are available. Our previous study revealed that the two gallated PA dimers epicatechin-3-gallate-(4β → 8, 2β → O → 7)-epicatechin-3-gallate (A-type ECG dimer) and epigallocatechin-3-gallate-(4β → 8, 2β → O → 7)-epigallocatechin-3 gallate (A-type EGCG dimer) inhibited the differentiation of 3T3-L1 cells significantly, while the nongallated epicatechin-(4β → 8, 2β → O → 7)-epicatechin (A-type EC dimer) and epicatechin-(4β → 8)-epicatechin (B-type EC dimer) showed little effect^17^, suggesting that the presence of the gallate moiety within the structure of PA dimers was also very important for their bioactivity.

How to explain the highly structure-dependent activity of PAs, as well as the great contribution of the gallate moiety to the bioactivity of PAs? Because many cellular processes such as cell signaling are membrane-dependent, increasing studies indicated that the different ability of PAs to interact with the membrane might give a partial explanation for their varied biological effects^18^,^19^,^20^. Although a few studies concerning the steric effects on the affinity of monomeric catechins to model lipid bilayers are available^21^, current data obtained with traditional techniques such as NMR, DSC methods could only provide information on a single time point of overall interacting state, but not a detailed molecular-level understanding of compounds-membrane interactions. Furthermore, PA oligomers and monomers differed greatly on their structural properties such as molecular size, DP, polarity, molecular topological surface area (tPSA) and spatial conformation etc. Therefore, the membrane interaction behaviors and modes of PA monomers may differ significantly from that of the PA oligomers. However, up to now, to our best knowledge, studies concerning the membrane interaction modes and behaviors of structural different PA oligomers are unavailable. Therefore, studies on the membrane interaction modes and behaviors of structural different PA oligomers are necessary.

Molecular dynamics (MD) simulation is a unique technique used in recent years for studying the dynamics of biological systems. It can provide quantitative atomic level molecular and thermodynamic descriptions of ligand-receptor interactions, and enable free space (atomistic) and time (sub-picosecond) resolutions, thus being a powerful complementary tool for elucidating biological mechanisms of bioactive compounds^22^,^23^. In present study, we examined and compared the interaction modes of the four structurally different PA dimers with mixed POPC/POPE lipid bilayer using MD methods. Our study can give insights into the interactions between PA dimers and bio-membranes at the atomic level, which can provide a foundation for elucidating the structure-activity relationship, as well as the underlying mechanisms of the bioactivities of PA oligomers.

## Results and Discussion

### Comparison of the orientation and location of the dimers on the lipid bilayer

The dynamic binding process of dimers to bilayer surface was firstly examined. Initially, the four PA dimers were inserted in the aqueous phase randomly. When MD started, the PA dimers quickly diffused and bound to the bilayer surface. The dynamic binding process and their relative position on the lipid bilayer were examined firstly. The center-of-mass (COM) molecular trajectories for the four dimers were showed in Supplementary Figure S1. During the simulation, all dimers showed a tendency to diffuse to the bilayer surface, but the time required for the dimers to bind to the bilayer surface and the distance between the dimers and bilayer surface varied notably. A-type ECG dimer and A-type EGCG dimer (Supplementary Fig. S1a,b) diffused to lipid bilayer surface faster and located much closer to the bilayer surface compared to A-type EC dimer and B-type EC dimer (Supplementary Fig. S1c,d), indicating that the two gallated dimers possibly had a higher affinity to the bilayer and penetrated into the bilayer much easier than the two nongallated dimers. As for the two gallated dimers, A-type ECG dimer bonded to bilayer faster and was more closer to the bilayer surface than A-type EGCG dimer. However, the COM position is not a definitive measure of the penetration of the dimers into the bilayer, because parts of the molecule can be relatively far from the COM, depending on the configuration and orientation of the dimers on the membrane surface^24^.

Because the bilayer normal is parallel to the Z-axis, the distance along the z-axis of the dimers relative to the center of lipid bilayer was used as a metric to study its spatial distribution on the membrane. The atomic number density profiles of the dimers along the z-axis were showed in Supplementary Fig. S2. The 0 nm indicated the center of lipid bilayer and the symmetric black solid line denoted the position of P-atoms. Distribution of the number densities (dashed line) described the relative position of dimers. The relative distances of the four dimers to the lipid bilayer center were 2.17, 2.37, 2.46, 2.57 nm for A-type ECG dimer (**a**), A-type EGCG dimer (**b**), A-type EC dimer (**c**) and B-type EC dimer (**d**), respectively. It was clear that A-type ECG and EGCG dimers penetrated much deeper into the bilayer than A-type EC and B-type EC dimers. And A-type ECG dimer penetrated about 0.2 nm more deep into the bilayer center than A-type EGCG dimer. The result agreed with that of COM trajectories well.

**Figure 1:**
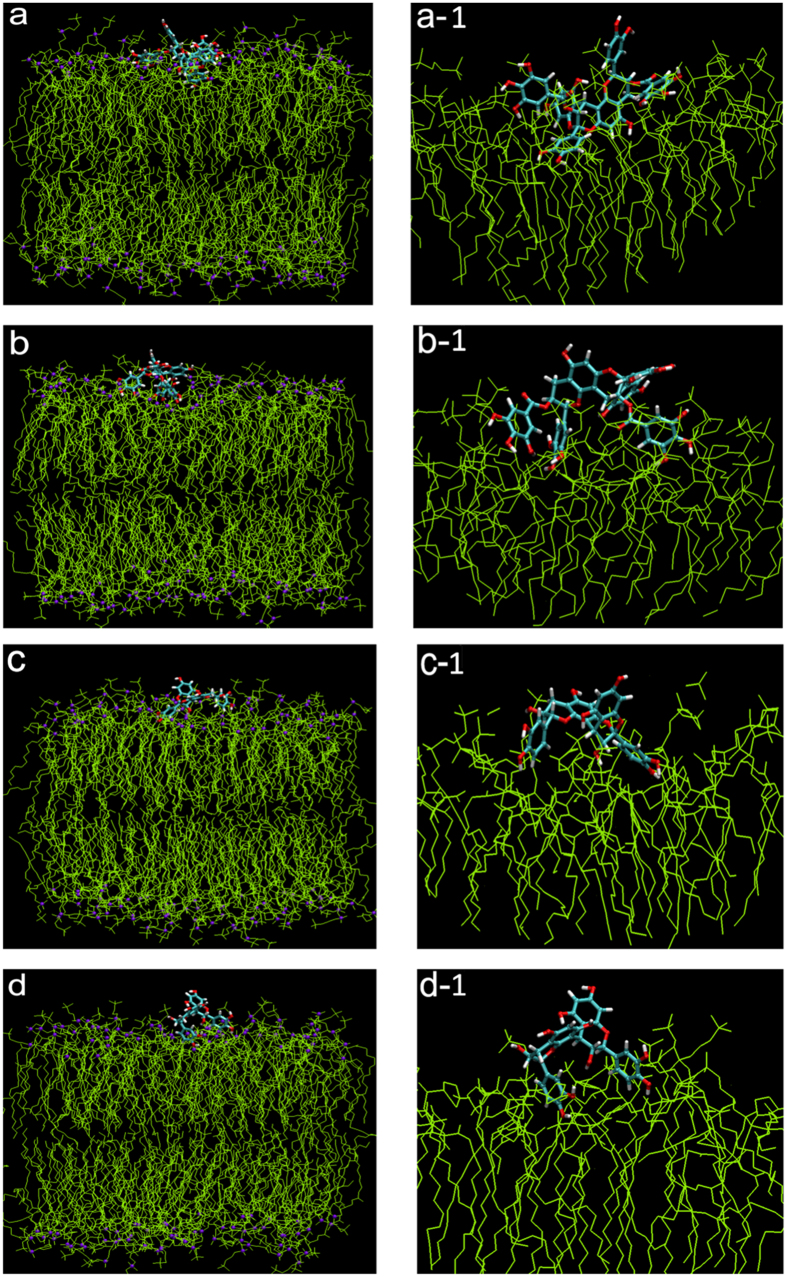
Snapshots of plain MD simulations characteristic to the location of (**a**) A-ECG dimer, (**b**) A-EGCG dimer, (**c**) A-EC dimer, (**d**) B-EC dimer and a-1, b-1, c-1, d-1 were the enlarged view of A-ECG dimer, A-EGCG dimer, A-EC dimer and B-EC dimer, respectively. The front views were gained by Viewer VMD 1.9.2. The line and ball model (yellow line for POPC and POPE lipids, violet ball for surface P-atoms) was used for membrane and bond model (cyan) for dimers. Water molecules were not represented for the sake of clarity. Each snapshot was chosen to be representative of the average location and orientation of the dimers.

We further investigated the equilibrium binding configurations and location of the four dimers on the bilayer. The last 50 ns trajectory was averaged to represent the equilibrium configurations of the dimers. As shown in Fig. 1, A-type ECG dimer (Fig. 1a,a-1) and A-type EGCG dimer (Fig. 1b,b-1) showed more extended conformations on the surface and they interacted strongly with the bilayer. A-type EGCG dimer was more elongated than A-type ECG dimer. In both dimers, ring B’ inserted deeply into the bilayer, while rings A/A’ bound on the surface of the lipid bilayer. The two gallate moieties (rings g1, g2) within both dimers inserted deeply into the lipid bilayer (Fig. 1a-1 and b-1 were enlarged snapshot of A-type ECG and EGCG dimers on the bilayer surface, separately). However, for A-type EC dimer, rings B and C’ predominantly interacted with the bilayer surface, while A/C/B rings remained in the water phase (Fig. 1c,c-1). As for B-type EC dimer (Fig. 1d,d-1), only a limited contacting sites were observed. The B and B’ rings contacted the bilayer surface, forming an inverted “V” shape, while other rings kept away from the bilayer. This data suggested that both A-type ECG and EGCG dimers could not only adsorb onto the surface of the bilayer, but also permeate deeply into the inside of the lipid bilayer, while A-type and B-type EC dimers only contacted with the surface with limited sites.

Compared with A-type EC dimer, a distinct molecular feature of A-type ECG and EGCG dimers is the presence of two additional gallate moieties. The higher affinity and deeper penetration of A-type ECG and EGCG dimers to the bilayer than that of A-type EC dimer indicated that the gallate moieties played a vital role in the membrane interaction of PAs. Our results were in line with that of Kajiya *et al*.^21^, who proved gallated catechins exerted stronger affinity for membrane than non-gallated catechins by fluorescent methods. However, simulations’ results in a pure POPC lipid bilayer model indicated that, unlike the nongallated EC molecules, the larger gallated ECG and EGCG molecules being absorbed into the bilayer harder than EC molecules while they were firstly in a aggregated state^25^. This suggested that the membrane behaviors of PA monomers differed significantly from that of PA dimers. In addition, the different membrane behavior of A-type EC and B-type EC dimers indicated that except for the gallate moieties, other structural feature such as the molecular size, the linkage type of interflavan bonds may also affect the membrane behavior of PAs greatly^26^.

### Hydrogen bond and free energy aspects of the dimer-bilayer interactions

Because the aromatic rings of PAs contain many hydroxyl substituents, hydrogen bonds (H-bonds) formed between the hydroxyl groups with the lipid oxygens might play an important role in the orientation and location of PAs on the bilayer surface^25^,^26^,^27^. To obtain insights into the different membrane behaviors of the four dimers, a detailed analysis of the H-bonds between the PA dimers and the lipids was performed. During the whole trajectories, a majority of the hydroxyl donors were engaged in H-bonds with either the bilayer or water. As demonstrated in Supplementary Fig. S3a-d, the dimers firstly formed H-bonds only with water, and as the dimers approached the bilayer, H-bonds were also formed between the dimers with lipid oxygens. The H-bond acceptors in bilayer and water seemed to compete for the H-bond donors in the dimers, as an increase of the H-bonds formed between dimers with bilayer paralleled with a decrease of H-bonds with water. For example, the H-bonds formed by A-type EGCG dimer increased from 0 to 8 with lipid bilayer, while it decreased from 25 to 15 with water (Supplementary Fig. S3b).

The average H-bonds formed with lipid bilayer was calculated to be 5.46 ± 0.31, 7.17 ± 0.26, 3.65 ± 0.25, 2.41 ± 0.24 for A-type ECG dimer, A-type EGCG dimer, A-type EC dimer and B-type EC dimer, respectively. The results suggested that the gallated dimers could form about 2 times more H-bonds with lipid bilayer than nongallated dimers. The tendency was in agreement with the reports by Sirk *et al*.^24^, who observed that TF3, 3′G (theaflavin with two gallate moiety) formed 3.26 average H-bonds with POPC/POPE lipid bilayer, while TF and TF3G (theaflavin with one gallate moiety) only formed 1.41, 1.29 average H-bonds, respectively. The presence of two gallate moieties allowed a configuration on the bilayer that efficiently forms H-bonds; a similar configuration was not possible in A-type EC dimer and B-type EC dimer. This result was consistent with their binding configuration on the bilayer surface well, and confirmed that the gallate ester moieties also played a vital role in the membrane interaction of PA dimers. The present analysis considered two cases: (i) dimer hydroxyl groups as H-donors and lipid oxygen groups as H-acceptors, and (ii) ethanolamine groups in POPE as H-donors and dimer oxygen atoms as H-acceptors. To test whether there was any preference of dimers for interacting with one of the two lipid types, the H-bonds formed separately with the POPC and POPE were calculated. As demonstrated in Supplementary Table S1. All dimers showed a tiny preference for lipid POPE than for POPC (they formed about 8–20% more H-bonds with POPE), but the preference was not significant (p > 0.05). It was possible that the ethanolamine groups in POPE exerted an additional contribution for the H-bonds formation.

**Figure 2:**
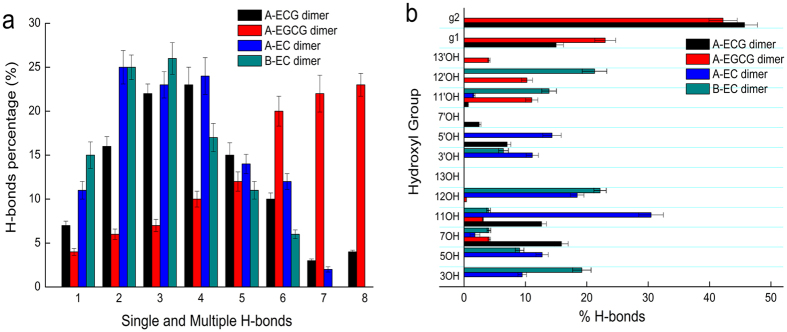
(**a**) Percentage of single and multiple H-bonds formed by the four dimers with POPC/POPE lipid bilayer over the 100 ns simulation. (**b**) H-bonds formed by individual hydroxyl groups and lipid oxygens. See Fig. 7 for the labeling of lipid oxygen atoms in POPC, POPE and hydroxyl groups in dimers. g1, g2 represented the gallate moiety in the structure of A-type ECG and EGCG dimers. Simulations were replicated five times independently. All values were represented as mean ± SD.

Because multiple hydroxyl groups could act as H-bond donors at the same time, simultaneous H-bonds formed by each dimer during the simulation were also considered. A-type and B-type EC dimers most likely formed one to three H-bonds on the surface of bilayer, with three H-bonds being dominant. However, when A-type ECG and EGCG dimers were absorbed to the bilayer, they could generate eight H-bonds. Multiple H-bonds (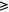
4) accounted for about 63 and 82% of the total number of H-bonds for interaction of A-type ECG and EGCG dimers with the bilayer, separately, significantly higher than that of A-type and B-type EC dimers with the values of 37 and 31%, separately (Fig. 2a). For the two gallated dimers, A-type EGCG dimer tended to form six to eight multiple H-bonds with the bilayer, while A-type ECG dimer was prone to form three to five multiple H-bonds with the bilayer, but six to eight multiple H-bonds could also be observed. Gallated dimers had a unique ability to form multiple H-bonds than nongallated dimers, probably due to the two additional gallate moieties in the structure.

**Figure 3:**
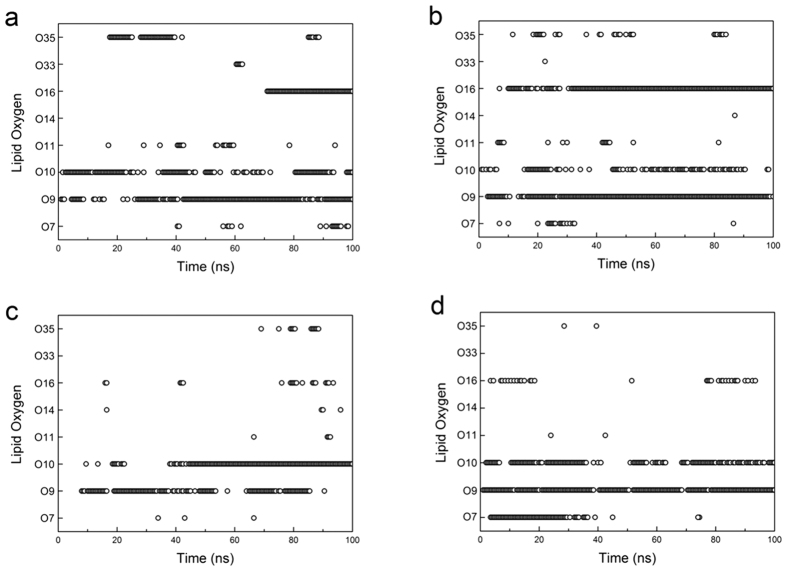
H-bonds formed between hydroxyl donors of (**a**) A-ECG dimer, (**b**) A-EGCG dimer, (**c**) A-EC dimer, (**d**) B-EC dimer and oxygen acceptors of the lipids during the 100 ns simulation. Each circle represented an H-bond formed between dimer and a specific lipid oxygen acceptor. See Fig. 7 for the labeling assignment of lipid oxygen atoms.

Figure 3 showed the H-bonds between the dimers to the respective lipid oxygen atoms (see Fig. 1 for naming assignment of the oxygen atoms of the POPC and POPE head-groups). Generally, dimers penetrating into the lipid bilayer showed predominant interactions with the deeper lipid oxygen atoms (O16, O33, and O35), whereas those adsorbing on the bilayer surface predominately formed H-bonds with the surface phosphate oxygen atoms (O7, O9, and O10). All dimers showed a strong preference for lipid surface acceptors O9 and O10. Additionally, A-type ECG and A-type EGCG dimer could also permeate into bilayer, forming H-bonds with the deeper inside lipid oxygen atoms O16 and O35, while A-type EC and B-type EC dimer was only prior to surface oxygen atoms O9 and O10.

The frequency and distribution of H-bonds formed by each hydroxyl group in dimers were shown in Fig. 2b. As for A-type ECG dimer and A-type EGCG dimer, the gallate moiety g1/g2 accounted for nearly 66% and 65% of the total H-bonds, separately. But for A-type EC dimer, B ring accounted for nearly 54% of the total H-bonds and B/B’ rings in B-type EC dimer formed about 70% of the total H-bonds. The data suggested that the two gallate moieties were the dominant groups forming H-bonds with lipid bilayer in A-type ECG dimer and A-type EGCG dimer, while B/B’ rings were the main H-donors in A-type EC dimer and B-type EC dimer.

The number of H-bonds formed between the dimers and bilayer depended not only on the number of both H-bond donors and acceptors, but also on the distance between the H-donors and H-acceptors. The presence of two gallate moieties could enhance their binding with the bilayer by providing more efficient hydroxyl groups for forming more simultaneous H-bonds. In addition, it allowed a special spatial configuration with the main skeleton structure outstretching on the surface and the gallate side chain permeating into the inside of the lipid bilayer, which permitted H-bonds formed with both surface and inside oxygens efficiently. However, the nongallated A-type EC dimer and B-type EC dimer seemed to predominantly form H-bonds in B ring. The difference in the frequency and distribution of H-bonds formed among the hydroxyl groups in dimers and lipid oxygens could give a good explanation for the more average and simultaneous H-bonds formed between the two gallated dimers with lipid bilayer than that of the nongallated dimers.

Apart from H-bonds interaction, other interactions such as van der Waals, electrostatic, polar solvation energy and non-polar solvation energy also contributed to the interaction of PAs with bilayer. The overall binding free energies, which took all the interaction forces into account, was a more comprehensive measurement of binding affinity of ligands to receptors^28^. According to previous study, the higher negative of the binding free energies values, the stronger affinity of the ligand to the receptor^29^. To provide further insight into the interactions between dimers with lipid bilayer, the binding free energy for the dimer-bilayer complex was evaluated using MM/PBSA method. Snapshots were extracted at every 100 ps from the 80–100 ns MD trajectory. The binding free energy obtained from the MM/PBSA calculation of the dimers-bilayer complex was listed in Table 1. It was clear that A-type EGCG dimer possessed the lowest binding free energy, followed by A-type ECG dimer. And the highest free energy value was observed in B-type EC dimer. The values of binding free energy of A-type EGCG and ECG dimers were about two to three times lower than that of A-type and B-type EC dimers, indicating the higher affinity of the former to the bilayer than the later. For the two gallated dimers, A-type EGCG dimer which had two more hydroxyl groups, possessed 30% higher binding free energy than A-type ECG dimer. Additionally, all dimers showed a tiny lower binding free energies with lipid POPE than with POPC (about lower 1–18 KJ/mol) (Supplementary Table S2), suggesting that the dimers had a relative higher affinities to lipid POPE than POPC. The result was in consistent with their H-bonds preference. Possibly the positive charge of the ethanolamine groups in POPE enhanced their affinity and interaction with the bilayer. The MM/PBSA method also permitted to decompose the overall binding free energy into several basic interaction forces, thereby providing more insights into the PA dimers-bilayer binding process. As shown in Table 1, both van der Waals and electrostatic interactions of A-type EGCG and ECG dimers with bilayer were higher than that of A-type and B-type EC dimers, suggesting that van der Waals and electrostatic interactions showed great contribution to the high affinity of A-type EGCG and ECG dimers to the bilayer. The electrostatic interactions of A-type EGCG dimer was about 33% lower than that of A-type ECG dimer, resulting in a lower binding free energy with the bilayer than that of A-type ECG dimer. Taken together, the more H-bonds, as well as the stronger van der Waals and electrostatic interactions of A-type EGCG and ECG dimers with bilayer than that of A-type and B-type EC dimer, could explain, at least in part, their dramatic differences in the affinity to lipid bilayer. The comparison of free energies with experimental values would be useful, but unfortunately, we did not obtain the experimental data for comparison of free energies. In our previous experimental studies, the affinity of the dimers for the synthetic liposome *in vitro* was indirectly determined by fluorescence spectroscopy and differential scanning calorimetry methods. We found that the results of MM/PBSA calculations were agreed well with our previous experimental results.

**Figure 4:**
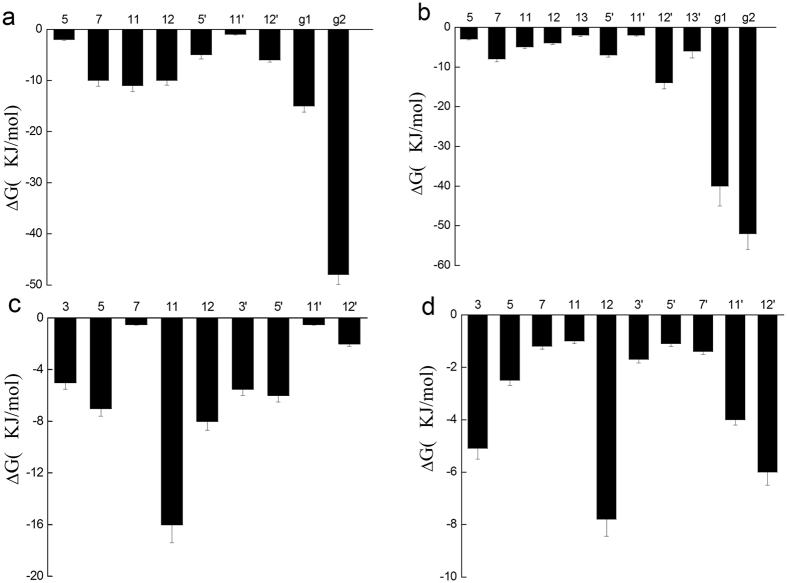
Binding free energy contribution of each hydroxyl group of (**a**) A-ECG dimer, (**b**) A-EGCG dimer, (**c**) A-EC dimer, (**d**) B-EC dimer in the dimer-bilayer complex. The hydroxyl group number and the gallate moiety g1, g2 was represented and labelled as Fig. 7. Simulations were replicated five times independently. All values were represented as mean ± SD.

According to previous study, the different hydroxyl groups in catechins have different polarity and electronic properties^30^,^31^. In order to figure out the contribution of each hydroxyl group to the total free energy of the dimer-bilayer complex, we decomposed the free energy. As shown in Fig. 4a–d, although all hydroxyl groups contributed to the binding free energies, the free energy contribution of hydroxyl groups differed notably. Compared to A-type EC and B-type EC dimers, two gallate moieties (g1/g2) in A-type ECG and EGCG dimers (Fig. 4a,b), provided a 60% additional contribution to the total free energies, except for the same levels of contribution generating by other hydroxyl groups in A/B rings with the value of about −10 KJ/mol for each hydroxyl group (Fig. 4c,d). However, there were also some differences between the two gallated dimers. In A-type ECG dimer, the gallate moiety g2 was much important than g1, but in A-type EGCG dimer, the two gallate moiety seemed to be equally important. For other hydroxyl groups, the contribution of 7, 11, 12-position hydroxyl groups in A-type ECG dimer relatively great, while in A-type EGCG dimer, the 7, 12′-position hydroxyl groups was more important. These differences might result from their orientations on the lipid bilayer. These data suggested that the two gallate moieties in A-type ECG and EGCG dimers greatly enhanced their affinities to the lipid bilayer.

As a negative control, an ensemble of 100 ns simulations of the neutral POPC lipid bilayer was also performed. The affinity of the four dimers to the POPC bilayer was analyzed and compared in terms of H-bonds and binding free energy. As shown in Supplementary Table S3, the H-bonds formed between the dimers and POPC bilayer calculated to be 4.23 ± 0.16, 4.57 ± 0.21, 2.98 ± 0.22 and 1.83 ± 0.14 for A-type ECG dimer, A-type EGCG dimer, A-type EC dimer and B-type EC dimer, respectively. The gallated dimers formed about 2 times higher H-bonds than the nongallated dimers. Though the H-bonds forming capacity of the dimers with POPC bilayer was lower than with mixed POPC/POPE bilayer, but the tendency (A-type EGCG dimer > A-type ECG dimer > A-type EC dimer > B-type EC dimer) was completely consistent. Previous reports by Sirk *et al*.^25^,^32^ also showed that EGCG formed more H-bonds with POPC or POPC/POPE lipid bilayer than ECG, EGC and EC. And the gallate moiety within gallated-catechins played an important role in their high affinity to lipid bilayer. Additionally, the binding free energies also verified the result. As shown in Supplementary Table S4, the binding free energies with POPC bilayer for A-type ECG dimer and A-type EGCG dimer was −116.77 ± 7.05, −121.81 ± 6.06 kJ/mol, separately, and for A-type EC dimer and B-type EC dimer was −49.62 ± 5.59, −33.43 ± 5.64 kJ/mol, separately. Both van der Waals and electrostatic interactions of A-type ECG and EGCG dimers with bilayer were higher than that of A-type EC and B-type EC dimers, indicating that van der Waals and electrostatic interactions contributed to their high affinity to the bilayer. But it is noteworthy that the electrostatic interactions with pure POPC bilayer of the dimers were lower than that of with POPC/POPE bilayer. The differences of the binding free energies between the four dimers in POPC bilayer were not as significant as that of in POPC/POPE bilayer (Table 1).

### Membrane structure aspects of the interaction

**Figure 5:**
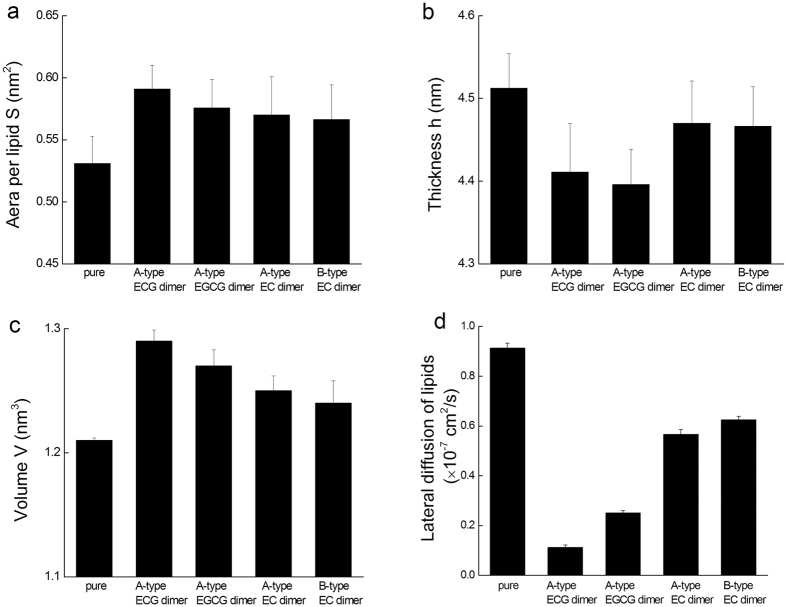
Effects of the four dimers on POPC/POPE lipid bilayer structural properties. (**a**) Area per lipid, (**b**) Bilayer thickness, (**c**) Volume per lipid and (**d**) Lateral diffusion of lipids. Simulations were replicated five times independently. All values were represented as mean ± SD.

Our previous study showed A-type ECG dimer and A-type EGCG dimer greatly affected the fluidity, hydrability and permeability of 3T3-L1 cells membrane^17^. Hence, effects of PA dimers on bilayer structure were further investigated. Figure 5a–d showed the area per lipid S, membrane thickness h, volume of per lipid V and lateral diffusion coefficient of lipids *K*_*d*_ in different simulation systems. These parameters are valuable metrics verifying the membrane fluidity. In general, the PA dimers increased the values of S and V, but decreased the values of *h* and *K*_*d*_ of the bilayer, suggesting that the absorption of PA dimers onto the bilayer resulted in a lateral expansion but a longitudinal compression of the bilayer.

Moreover, the absorption of dimers onto bilayer rigidified the bilayer and consequently reduced its mobility. The results could be explained by that, after being absorbed, the dimers were trapped between the lipid molecules, so the mobility of the lipids was restricted for the strong dimer-bilayer H-bonds and electrostatic attraction. The magnitude of changes in membrane fluidity induced by dimers was in consistent with their membrane penetration. The deeper penetration of dimers into the bilayer, the larger decrease of the bilayer fluidity. In addition, the binding of the dimers on the bilayer surface with outstretching configuration also laterally stretched out the bilayer, thus resulting in a lateral expansion of the bilayer. The changes of bilayer structure induced by A-type ECG and EGCG dimers were more notable than that of A-type EC and B-type EC dimers. The difference was possibly due to the more stretched structure of the gallated dimers and the deeper penetration of the two gallate moieties into bilayer.

**Figure 6:**
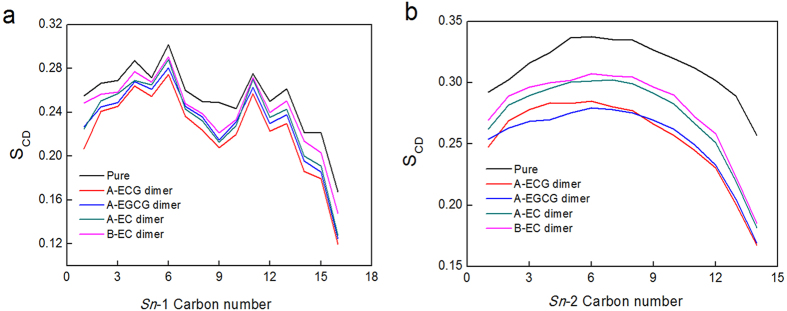
Effects of the four dimers on lipid tail order parameters of (**a**) *sn*-1 chain and (**b**) *sn*-2 chain of POPC/POPE lipid bilayer. See Fig. 1 for the labelling of *sn*-1 chain and *sn*-2 chain. Simulations were replicated five times independently. All values were represented as mean ± SD. Error bars were too low to be shown in the figure.

Additionally, the penetration of dimers into the bilayer center had substantial effects on the ordering of the lipid acyl chains. The order parameter (S_CD_) provided a quantitative measure of the alignment of the lipid tails. The higher value of S_CD_, the more ordered of the lipid tail chains^33^. Figure 6a–b showed the S_CD_ of the two lipid tails (*sn*-1 and *sn*-2) of the lipid bilayer in the different simulation systems. The value of S_CD_ of the two lipid tails with dimers was lower than that of purely hydrated membrane system, indicating the decrease of the lipid tails alignment. And interestingly, the sequence of effects on lipid tails ordering induced by dimers was in the order of A-type ECG dimer > A-type EGCG dimer > A-type EC dimer > B-type EC dimer. The membrane structure-perturbing potency of the dimers agreed well with their penetration depth into the bilayer (Supplementary Fig. S2), as well as their inhibitory effects on 3T3-L1 cell differentiation well^17^.

**Figure 7:**
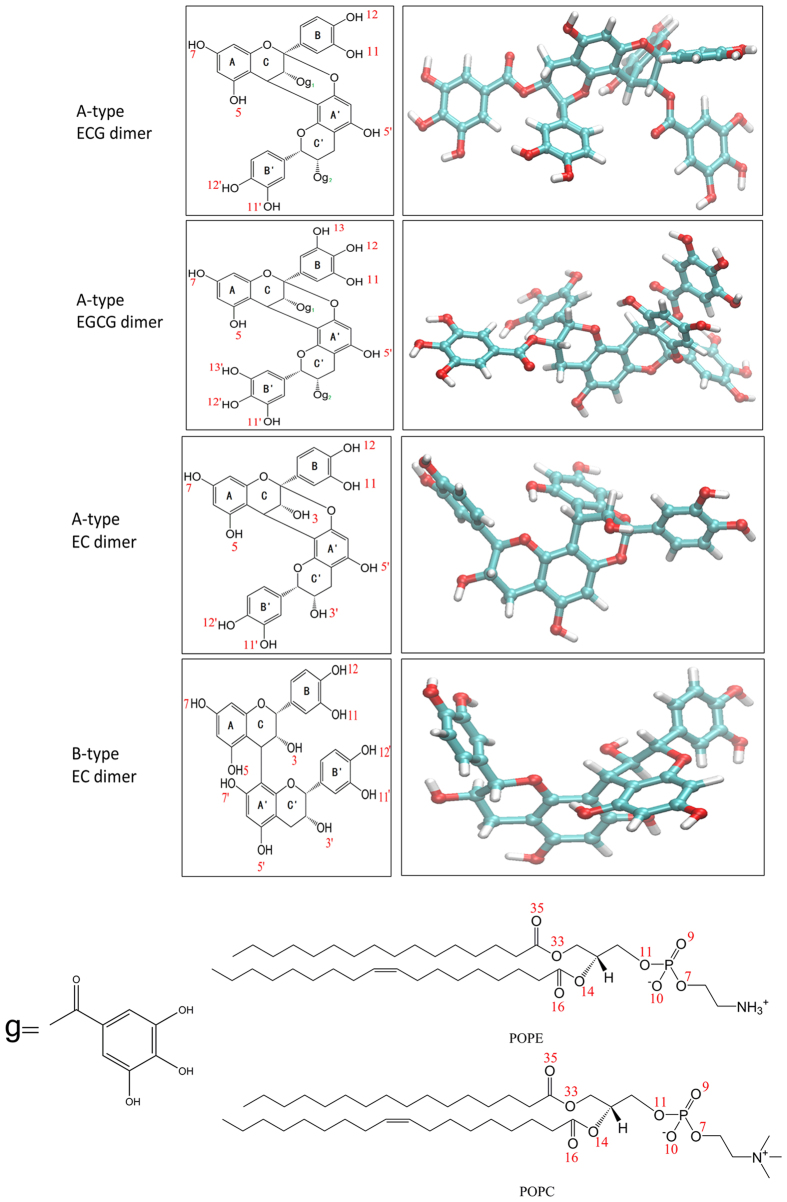
Planar chemical structure and spatial structure of epicatechin-3-gallate-(4β → 8, 2β → O → 7)-epicatechin-3-gallate (A-ECG dimer), epigallocatechin-3-gallate-(4β → 8, 2β → O → 7)-epigallocatechin-3-gallate (A-EGCG dimer), epicatechin-(4β → 8, 2β → O → 7)-epicatechin (A-EC dimer) and epicatechin-(4β → 8)-epicatechin (B-EC dimer) as well as chemical structures of lipid POPC/POPE molecule. The 2-D chemical structures were obtained by ChemDraw 14.0 and the 3-D structures were obtained by Viewer VMD 1.9.2.

It is known that the interaction with lipid bilayer of PAs is highly structure-dependent^34^,^35^,^36^. Compared with the monomers, PA dimers had more complex composition groups and steric configuration. The steric configuration was a crucial factor determining the potency of a compound interacting with the bilayer. According to the three-dimensional structures of the four dimers (Fig. 7), B-type dimers adopted a U-shaped conformation with the B/B’ rings from the terminal and extended units stacking, while the three A-type dimers were more elongated and rigid due to the additional 2β → O → 7 ether bond in the molecule. This facilitated that A-type dimers have a greater number of contacting sites with the membrane, thus resulting in a stronger interaction with the membrane.

In addition, the presence of two gallate moieties in the dimers enhanced the binding of the dimers with the bilayer greatly. This may be explained in three aspects. Firstly, the existing of two gallate moieties in A-type ECG and EGCG dimers provided more efficient OH donors for forming more H-bonds, thus enhancing their binding with the bilayer. Secondly, the presence of two gallate moieties in A-type ECG and EGCG dimers increased the hydrophobicity of the molecule, thus promoting the binding of the dimers with the hydrophobic region of the bilayer. Thirdly, the presence of two gallate moieties in the molecules resulted in a special spatial configuration of the dimers, allowing the main skeleton of the dimer to outstretch on the surface and the gallate side chains to permeate into the inside of the lipid bilayer easily. This led to more efficient contact of H-bonds donors and acceptors, as well as the hydrophobic domain of dimer with the inner lipophilic region of lipid bilayer.

We previously reported the significantly different inhibitory effects of the four dimers on 3T3-L1 preadipocytes differentiation^17^. We postulated that the significant different inhibitory effects of the four dimers on 3T3-L1 cell differentiation might be related to their distinct membrane disturbing potency.

## Conclusions

By extensive MD simulations of POPC/POPE lipid bilayer with four kinds of dimers, we found that the gallated PA dimers had higher affinities to the bilayer with much lower binding free energy compared with nongallated PA dimers. The gallated PA dimers penetrated deeper into the bilayer and formed more H-bonds with bilayer oxygen atoms, especially the inner oxygen atoms of the lipids, simultaneously, thus inducing stronger lateral expansion of the membrane and lipid tails disorder. The present results provided molecular insights into the interactions between PA dimers and bio-membranes and agreed with our experimental results well. These molecular interactions helped to elucidate the structure-function relationship of the PA dimers and provided a foundation for a better understanding of the underlying mechanisms of the bioactivities of PA oligomers.

## Materials and Methods

### Preparation of the PA dimers

Epicatechin-3-gallate-(4β → 8, 2β → O → 7)-epicatechin-3-gallate (A-type ECG dimer), epigallocatechin-3-gallate-(4β → 8, 2β → O → 7)-epigallocatechin-3-gallate (A-type EGCG dimer) were extracted from persimmon (*Diospyros kaki Thunb*. GongchengYueshi) and the proposed structure was elucidated in our earlier papers^37^, epicatechin-(4β → 8, 2β → O → 7)-epicatechin (A-type EC dimer) was obtained from peanut (Arachis hypogaea) red skins as we reported before^16^, epicatechin-(4β → 8)-epicatechin (B-type EC dimer) was isolated from Granny Smith apples (Malus domestica) and characterized by HPLC–MS/MS as we previously reported^38^. Their purity and identity were confirmed by HPLC and mass spectrometry. The purity of B-type EC dimer was confirmed by HPLC to be 96.57% using commercial procyanidin B1 as the standard, the purity of A-type dimer were analyzed by HPLC and calculated to be 95.72%, 95.56% and 97.28% for A-type ECG dimer, A-type EGCG dimer and A-type EC dimer, respectively, using procyanidin A2 as the standard. The planar 2D chemical structure obtained by ChemDraw 14.0 and the 3D structure obtained by Viewer VMD 1.9.2 of the four dimers were all shown in Fig. 1.

### Bilayer membrane model

Because phosphatidylcholine (PC) and phosphatidylethanolamine (PE) are the two dominant lipid species in biological membranes, lipid bilayers containing a 1:1 mixture of 1-palmitoyl-2-oleoyl-phosphati-dylcholine (POPC) and 1-palmitoyl-2-oleoyl-phosphatidylethanolamine (POPE) are often used as an ideal model for membrane interaction studies^39^. We hence chose the POPC/POPE (1:1) as the tested lipid bilayer model and the neutral POPC lipid bilayer as the negative control. The equilibrated POPC/POPE and pure POPC initial structure was obtained from Christian L^40^ and the topology file were downloaded from the Lipidbook server^41^. Force fields GROMOS53a6^42^ and Berger lipids parameters^43^ were used for phospholipids. Water used the transferable intermolecular potential 4 potential (TIP4P) model of Gromacs53A6. All-atom molecular structure and force field of the dimers were created using AmberTools^44^. The compatible of Amber force field and Berger lipid force field under Gromos53a6 has been improved in previous report^45^. The lipid bilayer model comprised 128 POPC and POPE molecules with 64 in each leaflet, surrounded by 4200 TIP4P water molecules. Before any calculation in the presence of the dimers, the lipid bilayer model was pre-equilibrated by 100 ns MD simulations. The stability of the equilibrated bilayer was confirmed from the area per lipid and S_CD_ of the lipid tails.

### Simulation details

All MD simulations and analysis for A-type ECG dimer, A-type EGCG dimer, A-type EC dimer and B-type EC dimer with a homogeneous mixed POPC/POPE lipid bilayer were carried out with GROMACS 5.0 software package^46^. Each system was solvated with a TIP4P water box with a margin of at least 10 Å from any edge of the box to any POPC/POPE or dimer atom. The box size was 6 × 6 × 10 nm and the dimer molecule was initially put in the center of water phase.

The linear constraint solver (LINCS) algorithm^47^ was applied to constrain all bonds in the lipids and dimers while the SETTLE algorithm^18^ was applied for water molecules. For Lennard-Jones and Coulombic interactions, a cutoff of 1.2 nm was applied, while electrostatic interactions were treated with the particle mesh Ewald (PME) method^48^ and a real-space cutoff of 1.2 nm. All systems were minimized through steepest descent minimization approach. The resulting systems were equilibrated for 1 ns with a time step of 2 fs in the NVT ensemble. The temperature was maintained at 310 K, corresponding to a liquid-crystalline state for the POPC/POPE bilayer through V-rescale thermostat with a coupling time constant of 0.1 ps. After NVT ensemble, the systems were further equilibrated for 1 ns with a time steps of 2 fs in the NPT ensemble. The pressure was maintained at 1 atm through Parrinello-Rahamn barostat with the coupling time constant to be 2.0 ps. The semi-isotropic pressure coupling scheme (isotropic in the x and y direction, but different in the z direction) with periodic boundary conditions were applied in all directions. For the production MD simulations, the barostat and thermostat parameters maintained the same as for the NPT ensemble. Snapshots were stored every 2 ps. The variation of root mean square deviation (RMSD) values of all systems were within 0.5 Å in the last 50 ns (Supplementary Fig. S4a–d), indicating the equilibrium of trajectories. So, the last 50 ns trajectory was used for production trajectory analysis.

### Analysis

The surfaces of the lipid bilayer were defined by the layer of phosphorus atoms (P-atoms) of the POPC/POPE head groups and were oriented parallel to the xy-plane. The simulation trajectories were analyzed using several auxiliary programs provided with the GROMACS 5.0 package. The programs included g_traj for calculating the center of mass (COM) trajectories of dimers, g_density for the distribution of atomic number density, as well as g_energy, g_hbond, g_msd and g_order, respectively.

The number of hydrogen bond (H-bond) (X—H···Y) was analyzed assuming the presence of a H-bond when (i) the distance between H-bond donor and acceptor d(X—Y) was lower than 3.5 Å and (ii) the angle H—X—Y was lower than 30°. After the whole system was well equilibrated, analysis of the distributions of position, orientation, and number of H-bonds was performed over the last 50 ns of MD simulations. The program g_hbond was applied to calculate the number of H-bonds between the dimers and lipid bilayer.

For the membrane structure analysis, the area per lipid was defined as the area of the xy-plane of the simulation box divided by the number of lipids per leaflet. The thickness of lipid bilayer h was defined as the distance between the average z-position of the P-atoms in the two layers. And the volume per lipid is defined as V = Sh/2. The box vectors can be easily extracted from the energy file using g_energy. The programs g_msd and g_order of GROMACS 5.0 were applied to calculate the lateral diffusion coefficients and lipid tail order parameters. Simulations of 100 ns for each dimer were performed five times independently, for a total of 20 simulations of 100 ns. The initial conditions (the position and thermal velocities) of the dimers were different for each run (The initial position of the dimers in the five repeat simulations was shown in Supplementary Fig. S5. We just took A-EC dimer as example). After analysis about the five trajectory of each dimer, we found the initial position/orientation and thermal velocity of the dimers did not affect their interaction with bilayer after the system equilibrium. Error bars of the results were calculated using software SPSS19.0 with five set of data. The results were expressed as the mean ± SD of five replications of each group. The data of H-bonds and free energies for the five repeat simulations of each dimer was shown in Supplementary Information.

Simulation trajectories were visualized, and all of the snapshots were prepared using the Viewer VMD 1.9.2^49^.

### Calculation and decomposition of binding free energy

The molecular mechanics Poisson Boltzmann surface area (MM/PBSA) method is a reliable and widely used method for binding free energies calculations from the snapshots of MD trajectory^28^,^50^,^51^. The binding free energy of a given ligand-receptor complex is calculated by summing up molecular mechanical energies, solvation energies, and vibrational entropies. Normal mode analysis supplements the MM/PBSA method to approximate more accurately the total free energy. In the present work, the binding free energies of the complexes between dimers and POPC/POPE lipid bilayer were analyzed by taking snapshots at an interval of 100 ps from 80 to 100 ns MD simulations during equilibrium phase, using g_mmpbsa tool of Gromacs^52^.

Particularly, the binding free energy of dimer-bilayer complex in solvent was expressed as:


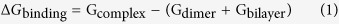


Where G_complex_ is the total free energy of the dimer-bilayer complex, G_dimer_ and G_bilayer_ are total energy of separated dimer and bilayer in solvent, respectively. The free energy for each individual G_complex_, G_dimer_ and G_bilayer_ were estimated by:


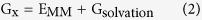


Where x is the dimer, bilayer or complex. E_MM_ is the average molecular mechanics potential energy in vacuum and G_solvation_ is free energy of solvation. The molecular mechanics potential energy was calculated in vacuum as following:


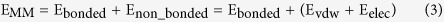


Where E_bonded_ is bonded interaction including of bond, angle, dihedral and improper interactions and E_non-bonded_ is non-bonded interactions consisting of van der Waals (E_vdw_) and electrostatic (E_elec_) interactions. ΔE_bonded_ is always taken as zero.

The solvation free energy (G_solvation_) was estimated as the sum of electrostatic solvation free energy (G_polar_) and apolar solvation free energy (G_non-polar_):


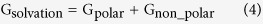


Where G_polar_ was computed using the Poisson-Boltzmann (PB) equation and G_non-polar_ estimated from the solvent-accessible surface area (SASA) as equation following:


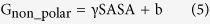


Where γ is a coefficient related to surface tension of the solvent and b is fitting parameter. The values of the contats are as follows:


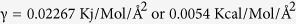



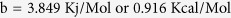


Per-residue free energy decomposition based on the MM/PBSA method was performed to address the contribution of each hydroxyl group to the binding free energies of the dimer-bilayer complex^50^.

## Additional Information

**How to cite this article**: Zhu, W. *et al*. Molecular Insight into Affinities of Gallated and Nongallated Proanthocyanidins Dimers to Lipid Bilayers. *Sci. Rep.* **6**, 37680; doi: 10.1038/srep37680 (2016).

**Publisher’s note:** Springer Nature remains neutral with regard to jurisdictional claims in published maps and institutional affiliations.

## Supplementary Material

Supplementary Information

## Figures and Tables

**Table 1:** Average MM/PBSA free energies of dimer-bilayer complexes calculated from the MD simulations performed in quintuplicate.

	A-ECG dimer	A-EGCG dimer	A-EC dimer	B-EC dimer
van der Waal energy (kJ/mol)	−218.76 ± 15.18	−232.86 ± 15.27	−151.58 ± 11.01	−135.77 ± 8.57
Electrostattic energy (kJ/mol)	−168.83 ± 19.27	−250.67 ± 11.46	−164.05 ± 7.12	−138.36 ± 9.31
Polar solvation energy (kJ/mol)	328.85 ± 17.46	346.48 ± 11.19	336.01 ± 12.71	306.33 ± 10.16
SASA energy (kJ/mol)	−62.60 ± 8.79	−42.30 ± 6.02	−71.10 ± 6.14	−63.11 ± 6.40
Binding energy (kJ/mol)	−121.34 ± 12.87	−179.35 ± 7.09	−50.72 ± 4.85	−30.61 ± 3.97

Results were shown as the mean + SD of five replications.
