# Supplementary material for: Molecular Insight into Affinities of Gallated and Nongallated Proanthocyanidins Dimers to Lipid Bilayers
Source: Sci Rep. 2016 Nov 22;6:37680. doi: 10.1038/srep37680 (PMC5118708; doi:10.1038/srep37680)
Supplement: Supplementary Information [file srep37680-s1.docx]

**Supplementary Information**

**Molecular Insight into Affinities of Gallated and Nongallated** **Proanthocyanidins Dimers to Lipid Bilayers**

Wei Zhu^1^, Le Xiong^2^, Jinming Peng^1^, Xiangyi Deng^1^, Jun Gao^2*^, Chun-mei Li^1^, ^3*^

^1^College of Food Science and Technology, Huazhong Agricultural University, Wuhan, China, 430070

^2^Hubei Key Laboratory of Agricultural Bioinformatics, College of Informatics, Huazhong Agricultural University, Wuhan, China, 430070

^3^Key Laboratory of Environment Correlative Food Science (Huazhong Agricultural University), Ministry of Education

*Corresponding author: Chunmei Li (Tel: 86-27-87282966; Fax: 86-27-87282966; E-mail: lichmyl@mail.hzau.edu.cn); Jun Gao (E-mail: gaojun@mail.hzau.edu.cn).

**Figure S1** The center of mass (COM) trajectory of (a) A-ECG dimer, (b) A-EGCG dimer, (c) A-EC dimer, (d) B-EC dimer in the POPC/POPE lipid bilayer system along the 100 ns simulation. The solid blue lines represented the average position of the phosphorus atoms in the POPC/POPE head-groups. Position zero corresponded to the middle of the aqueous phase. Regions above and below the solid blue lines corresponded to the lipid bilayer.

**Figure S2** Relative distribution of the atomic number densities (dashed line) describing the position of (a) A-ECG dimer, (b) A-EGCG dimer, (c) A-EC dimer, (d) B-EC dimer. The black curves denoted the position of phosphorus atoms.

**Figure S3** Hydrogen bonds of dimers (a) A-ECG dimer, (b) A-EGCG dimer, (c) A-EC dimer, (d) B-EC dimer simultaneously formed with water (red) and lipid oxygen (black) during the course of 100 ns simulation.

**Figure S4** The root mean square deviation（RMSD）of the four simulation systems. (a) A-ECG dimer, (b) A-EGCG dimer, (c) A-EC dimer, (d) B-EC dimer within the POPC/POPE lipid bilayer system.

**
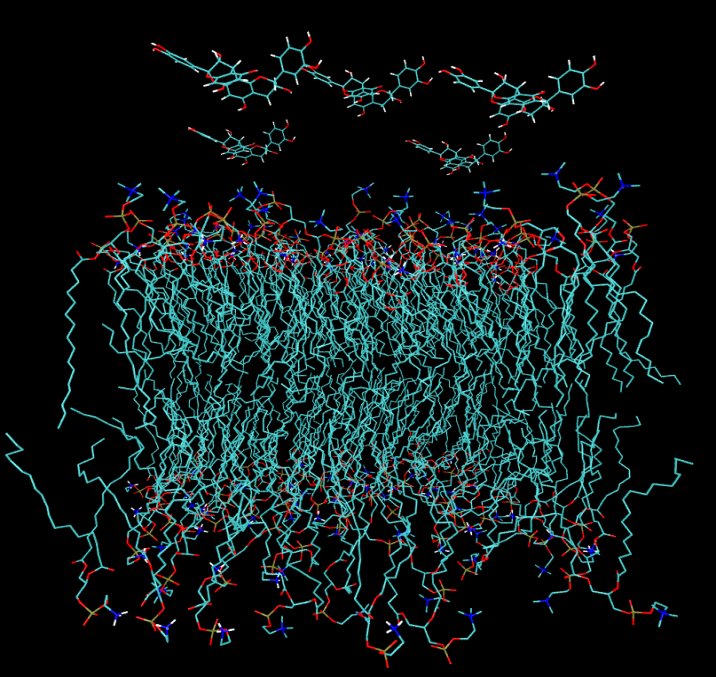
**

| Times | X | Y | Z |
| --- | --- | --- | --- |
| 1 | 1.5 | 1.5 | 7.5 |
| 2 | 1.5 | 4.5 | 7.5 |
| 3 | 3 | 3 | 7.5 |
| 4  5 | 4.5  4.5 | 1.5  4.5 | 7.5  7.5 |

**Figure S5** The initial positions of the dimers for the five independent repeat simulations (A-EC dimer was taken as example). X, Y, Z represent the coordinate value along the x, y, z-axis, respectively.

**Table S1** The average hydrogen bonds formed between the four dimers and POPC or POPE during the last 50 ns molecular dynamics simulation. Simulations were replicated five times independently. All values were represented as mean±SD.

|  | POPC | POPE | Total |
| --- | --- | --- | --- |
| A-ECG dimer | 2.31±0.12 | 3.15±0.23 | 5.46±0.51 |
| A-EGCG dimer | 3.12±0.23 | 4.05±0.37 | 7.17±0.68 |
| A-EC dimer | 1.62±0.11 | 2.03±0.21 | 3.65±0.41 |
| B-EC dimer | 1.18±0.12 | 1.23±0.16 | 2.41±0.26 |

**Table S2** Average MM/PBSA free energies of dimer interacting with lipid POPC and POPE calculated from the MD simulations performed in quintuplicate. All values were represented as mean±SD.

|  | A-ECG dimer | A-EGCG dimer | A-EC dimer | B-EC dimer |
| --- | --- | --- | --- | --- |
| POPC  POPE | -53.87±3.14  -67.47±5.18 | -80.28±10.76  -99.07±8.29 | -23.59±2.28  -27.13±2.17 | -14.27±1.26  -16.64±1.88 |
| Total (kJ/mol) | -121.34±11.91 | -179.35±10.23 | -50.72±5.29 | -30.91±3.18 |

**Table S3** The average hydrogen bonds formed between the four dimers with the neutral control POPC lipid bilayer during the last 50 ns trajectory of the five repeat molecular dynamic simulations. All values were represented as mean±SD.

|  | 1 | 2 | 3 | 4 | 5 | Average |
| --- | --- | --- | --- | --- | --- | --- |
| A-ECG dimer | 3.99 | 4.21 | 4.42 | 4.32 | 4.22 | 4.23±0.16 |
| A-EGCG dimer | 4.37 | 4.69 | 4.88 | 4.41 | 4.49 | 4.57±0.21 |
| A-EC dimer | 2.81 | 3.28 | 2.74 | 2.89 | 3.15 | 2.98±0.22 |
| B-EC dimer | 1.97 | 2.16 | 1.72 | 2.04 | 1.81 | 1.83±0.14 |

**Table S4** Average MM/PBSA free energies of dimers interacting with the neutral control POPC lipid bilayer calculated from the MD simulations performed five times independently. All values were represented as mean±SD.

|  | A-ECG dimer | A-EGCG dimer | A-EC  dimer | B-EC  dimer |
| --- | --- | --- | --- | --- |
| van der Waal energy  (kJ/mol) | -221.36±14.29 | -245.24±10.32 | -182.52±6.91 | -138.77±8.97 |
| Electrostattic energy  (kJ/mol) | -148.13± 11.18 | -116.80±8.14 | -100.81±7.98 | -120.22±6.53 |
| Polar solvation energy  (kJ/mol) | 321.35±5.27 | 325.17±7.12 | 317.24±7.19 | 306.84±9.42 |
| SASA energy  (kJ/mol) | -68.63±12.05 | -85.20±4.97 | -83.81±1.92 | -81.63±7.64 |
| Binding energy  (kJ/mol) | -116.77±7.05 | -121.81±6.06 | -49.62±5.59 | -33.43±5.64 |

**Table S5** The data of free energy of the four dimers with neutral control POPC lipid bilayer for the five repeat simulations.

**Table S5a** Free energy of A-ECG dimer for the five repeat simulations

|  | 1 | 2 | 3 | 4 | 5 | Average |  |
| --- | --- | --- | --- | --- | --- | --- | --- |
| van der Waal energy  (kJ/mol) | -234 | -231 | -225 | -198 | -219 | -221.36±14.29 |  |
| Electrostattic energy  (kJ/mol) | -160 | -156 | -131 | -148 | -146 | -148.13± 11.18 |  |
| Polar solvation energy  (kJ/mol) | 326 | 319 | 316 | 318 | 328 | 321.35±5.27 |  |
| SASA energy  (kJ/mol) | -54 | -57 | -76 | -79 | -77 | -68.63±12.05 |  |
| Binding energy  (kJ/mol) | -122 | -125 | -116 | -107 | -114 | -116.77±7.05 |  |

**Table S5b** Free energy of A-EGCG dimer for the five repeat simulations

|  | 1 | 2 | 3 | 4 | 5 | Average |  |
| --- | --- | --- | --- | --- | --- | --- | --- |
| van der Waal energy  (kJ/mol) | -231 | -252 | -239 | -257 | -246 | -245.24±10.32 |  |
| Electrostattic energy  (kJ/mol) | -121 | -108 | -227 | -109 | -119 | -116.80±8.14 |  |
| Polar solvation energy  (kJ/mol) | 315 | 326 | 324 | 355 | 326 | 325.17±7.12 |  |
| SASA energy  (kJ/mol) | -79 | -87 | -82 | -86 | -92 | -85.20±4.97 |  |
| Binding energy  (kJ/mol) | -181 | -121 | -124 | -117 | -131 | -121.81±6.06 |  |

**Table S5c** Free energy of A-EC dimer for the five repeat simulations

|  | 1 | 2 | 3 | 4 | 5 | Average |  |
| --- | --- | --- | --- | --- | --- | --- | --- |
| van der Waal energy  (kJ/mol) | -181 | -184 | -186 | -189 | -171 | -182.52±6.91 |  |
| Electrostattic energy  (kJ/mol) | -104 | -91 | -96 | -101 | -112 | -100.81±7.98 |  |
| Polar solvation energy  (kJ/mol) | 321 | 308 | 312 | 319 | 326 | 317.24±7.19 |  |
| SASA energy  (kJ/mol) | -86 | -81 | -85 | -83 | -84 | -83.81±1.92 |  |
| Binding energy  (kJ/mol) | -50 | -48 | -55 | -54 | -41 | -49.62±5.59 |  |

**Table S5d** Free energy of B-EC dimer for the five repeat simulations

|  | 1 | 2 | 3 | 4 | 5 | Average |  |
| --- | --- | --- | --- | --- | --- | --- | --- |
| van der Waal energy  (kJ/mol) | -138 | -152 | -143 | -126 | -133 | -138.77±8.97 |  |
| Electrostattic energy  (kJ/mol) | -114 | -121 | -126 | -127 | -113 | -120.22±6.53 |  |
| Polar solvation energy  (kJ/mol) | 308 | 314 | 317 | 301 | 294 | 306.84±9.42 |  |
| SASA energy  (kJ/mol) | -86 | -71 | -91 | -82 | -78 | -81.63±7.64 |  |
| Binding energy  (kJ/mol) | -30 | -30 | -43 | -34 | -30 | -33.43±5.64 |  |

**Table S6** The hydrogen bonds formed between the four dimers with POPC/POPE lipid bilayer during the last 50 ns trajectory of the five repeat simulations. All values were represented as mean±SD.

|  | 1 | 2 | 3 | 4 | 5 | Average |
| --- | --- | --- | --- | --- | --- | --- |
| A-ECG dimer | 5.19 | 5.33 | 5.86 | 5.72 | 5.22 | 5.46±0.31 |
| A-EGCG dimer | 6.98 | 7.18 | 6.88 | 7.31 | 7.52 | 7.17±0.26 |
| A-EC dimer | 3.81 | 3.38 | 3.74 | 3.92 | 3.38 | 3.65±0.25 |
| B-EC dimer | 2.27 | 2.36 | 2.72 | 2.59 | 2.11 | 2.41±0.24 |

**Table S7** The data of free energy of the four dimers with POPC/POPE bilayer for the five repeat simulations.

**Table S7a** Free energy of A-ECG dimer for the five repeat simulations

|  | 1 | 2 | 3 | 4 | 5 | Average |  |
| --- | --- | --- | --- | --- | --- | --- | --- |
| van der Waal energy  (kJ/mol) | -229 | -237 | -216 | -199 | -212 | -218.76±15.18 |  |
| Electrostattic energy  (kJ/mol) | -173 | -159 | -141 | -181 | -190 | -168.83± 19.27 |  |
| Polar solvation energy  (kJ/mol) | 329 | 337 | 314 | 338 | 326 | 328.85±17.46 |  |
| SASA energy  (kJ/mol) | -55 | -67 | -76 | -58 | -57 | -62.60±8.79 |  |
| Binding energy  (kJ/mol) | -128 | -126 | -119 | -100 | -133 | -121.34±12.87 |  |

**Table S7b** Free energy of A-EGCG dimer for the five repeat simulations

|  | 1 | 2 | 3 | 4 | 5 | Average |  |
| --- | --- | --- | --- | --- | --- | --- | --- |
| van der Waal energy  (kJ/mol) | -213 | -229 | -245 | -251 | -226 | -232.86±15.27 |  |
| Electrostattic energy  (kJ/mol) | -254 | -242 | -259 | -237 | -261 | -250.67±11.46 |  |
| Polar solvation energy  (kJ/mol) | 332 | 349 | 354 | 359 | 338 | 346.48±11.19 |  |
| SASA energy  (kJ/mol) | -46 | -50 | -35 | -43 | -38 | -42.30±6.02 |  |
| Binding energy  (kJ/mol) | -181 | -172 | -185 | -172 | -187 | -179.35±7.09 |  |

**Table S7c** Free energy of A-EC dimer for the five repeat simulations

|  | 1 | 2 | 3 | 4 | 5 | Average |  |
| --- | --- | --- | --- | --- | --- | --- | --- |
| van der Waal energy  (kJ/mol) | -156 | -168 | -146 | -149 | -139 | -151.58±11.01 |  |
| Electrostattic energy  (kJ/mol) | -157 | -159 | -174 | -169 | -162 | -164.05±7.12 |  |
| Polar solvation energy  (kJ/mol) | 318 | 349 | 346 | 338 | 329 | 336.01±12.71 |  |
| SASA energy  (kJ/mol) | -64 | -68 | -76 | -69 | -79 | -71.10±6.14 |  |
| Binding energy  (kJ/mol) | -59 | -46 | -50 | -49 | -51 | -50.72±4.85 |  |

**Table S7d** Free energy of B-EC dimer for the five repeat simulations

|  | 1 | 2 | 3 | 4 | 5 | Average |  |
| --- | --- | --- | --- | --- | --- | --- | --- |
| van der Waal energy  (kJ/mol) | -127 | -143 | -146 | -129 | -133 | -135.77±8.57 |  |
| Electrostattic energy  (kJ/mol) | -149 | -147 | -130 | -137 | -129 | -138.36±9.31 |  |
| Polar solvation energy  (kJ/mol) | 299 | 324 | 317 | 296 | 296 | 306.33±10.16 |  |
| SASA energy  (kJ/mol) | -56 | -68 | -71 | -62 | -58 | -63.11±6.40 |  |
| Binding energy  (kJ/mol) | -33 | -34 | -30 | -32 | -24 | -30.61±3.97 |  |
